# Sorting Olive Batches for the Milling Process Using Image Processing

**DOI:** 10.3390/s150715738

**Published:** 2015-07-02

**Authors:** Daniel Aguilera Puerto, Diego Manuel Martínez Gila, Javier Gámez García, Juan Gómez Ortega

**Affiliations:** 1 ANDALTEC, Plastic Technological Center, Martos, Jaén 23600, Spain; E-Mail: aguilera@andaltec.org; 2 Group of Robotics, Automation and Computer Vision, University of Jaén, Agrifood Campus of International Excellence (ceiA3), Jaén 23071, Spain; E-Mails: jggarcia@ujaen.es (J.G.G.); juango@ujaen.es (J.G.O.)

**Keywords:** olive classification, computer vision, automatic quality control

## Abstract

The quality of virgin olive oil obtained in the milling process is directly bound to the characteristics of the olives. Hence, the correct classification of the different incoming olive batches is crucial to reach the maximum quality of the oil. The aim of this work is to provide an automatic inspection system, based on computer vision, and to classify automatically different batches of olives entering the milling process. The classification is based on the differentiation between ground and tree olives. For this purpose, three different species have been studied (Picudo, Picual and Hojiblanco). The samples have been obtained by picking the olives directly from the tree or from the ground. The feature vector of the samples has been obtained on the basis of the olive image histograms. Moreover, different image preprocessing has been employed, and two classification techniques have been used: these are discriminant analysis and neural networks. The proposed methodology has been validated successfully, obtaining good classification results.

## 1. Introduction

The production of virgin olive oil is an important economic activity carried out in more than 30 countries. Currently, the main use of olives worldwide is to produce olive oil (about 88% of the total olive production). The main factor that affects the olive oil quality is the raw material: the olive fruit. As a result, olive quality is related to agronomic conditions and harvesting conditions. In [1], the influences of the cultivar, kind of soil, level of fertilization, harvest day and other significant parameters are stated.

Besides, the olive oil production process could affect the oil quality. Many research works have been carried out to determine the influence of the manufacturing process on olive oil quality. In [2], the authors compare different processes and extraction conditions and their influence on main olive oil quality parameters. This process consists of several phases. It depends on a great number of physical variables and presents different control setups, which are described in [3,4]. It is therefore a complex process.

One of the key factors of olive oil production is the correct reception and classification of olives before the milling, in order to produce the maximum quality level with the maximum process performance. In [5], the fruit reception is classified as one of the most important steps in the oil extraction process.

The first classification that could be done of fruits entering the mill is the differentiation between ground and tree olive, that is the fruit that has fallen to the soil and later has been picked up and olives that have been picked directly from the tree. This is because the fruit on the ground undergoes a series of changes that deteriorate the quality of the oil obtained. For example, the acidity increases when the time of remaining on the soil is longer. In addition to this, the organoleptic quality is affected; the tree oil is usually extra virgin, with a great fragrance and positive attributes; conversely, soil oil deteriorates to lampante virgin olive oil, with major defects and is unfit for direct consumption [6]. Olives coming from the ground also contain waste, such as soil, pieces of branches and some pebbles.

Some discriminatory features can be observed by looking at the olives harvested from tree and soil. These features are mainly related to the color and the morphological appearance of their skin. The olives picked from the tree are normally green, purple or black (according to the ripening index) [7] and smooth skinned. What is more, when the olives have remained on the ground for several days, they turn brownish due to the oxidation process, and their skin changes to wrinkled, because they start to dry. In addition, olive batches coming from the soil are less homogeneous than olives from the tree due to the fact that they have fallen at different moments and they have other elements, such as soil grains, pebbles or leaves.

Nowadays, in olive oil production, the quality evaluation of the incoming fruit batches depends on manual inspection, and an operator will decide if the batch is from the tree, the soil, mixed or even if it has other kinds of defects. Obviously, this selection is not often carried out properly, since it is subjected to personal criteria, and it is a complex decision to be made in a quick inspection. Later, depending on this previous classification, the oil extracted in the milling goes to different tanks classified by the quality. Olives should have been previously classified by the farmer, as is reported in [8], but sometimes, the fruit has been mixed in order to reduce the harvest cost. In [9], the authors expose the costs and profits in different kinds of olive fields. It is absolutely necessary to collect, transport and process fruits from soil and tree separately. Similarly, in [6], it is stated that a small amount of olives coming from soil can significantly modify the organoleptic characteristics of olive oil from the tree.

As soon as the olive lots have been separated, independent production lines should be used to manufacture the olive oil. In this way, it would be easy to keep the best possible olive oil quality until the end of the production process.

In this work, we propose an automatic classification methodology of olives based on computer vision. The aim of the classification is the differentiation between tree and soil olive lots for oil production, trying to minimize possible grading mistakes.

The structure of the paper is as follows. Firstly, the materials and the methods used for our experimentation are exposed. Secondly, some experimental results are shown. Finally, conclusions and future work are presented.

### 1.1. Background and Earlier Works

The increased expectations for food products of high quality and safety standards demand innovative techniques of food inspection in the production chains [10]. As a consequence, the number of research papers about non-invasive techniques for quality assessment have increased in the last few years, such as electronic noses [11], electronic tongues [12] and hyper-spectral imaging [13]. Computer vision is another one, and it has been used with learning techniques, like neural networks, statistical learning, fuzzy logic, decision tree or genetic algorithms [14].

The computer vision applications are widely extended as an application to check the quality of products in the food industry. For example, in [15], genetic algorithms combined with the nearest neighbor method were used for the discrimination of four kinds of seeds using artificial vision. In [16], the authors show a grading system based on image processing techniques, which was developed for automatically inspecting and grading flue-cured tobacco leaves. Apart from this, in [17], the authors used the histograms of the red and green channels to classify dates according to their maturity index. Furthermore, in [18], the researchers developed a machine for the automatic sorting of pomegranate arils. They used the information of red, green and blue channels. Their classification algorithm is a linear discriminating analysis. In addition, in [19], the researchers employed neural networks for grading beans according to their color, with a success of 90,6%. Likewise, in [20], we can find a way to estimate the antioxidant activity and anthocyanin content of sweet cherries during ripening, combining a vision system with an artificial neural network and adaptive neuro-fuzzy inference. In [21], the authors presented a technique using computer vision to detect disease stress in wheat, too.

The aforementioned technology has been used at different stages of the olive oil elaboration process. Previous works have been presented for the automatic classification of olive fruits using artificial vision. As an example, in [22], an automatic inspection system was developed for table olives. It was based on computer vision, which takes into account aspects, such as the color of the skin or the presence of defects on the surface of the olive. In this work, three algorithms were tested, Bayesian and PLSmulti-variant discriminant analysis and neural networks. The best result was obtained with the neural network, with an accuracy of over 90%. This work was focused on green olives, and three pictures of each olive are needed to find each defect in the complete olive's surface. In our case, it is enough to have one picture. On the other hand, the image processing was used to predict the olive oil content [23] using two prediction models based on linear regressions and artificial neural networks. Besides this, ANN and image analysis were employed to estimate the olive ripening index [24]. In [25], a method of classifying olives for oil production was developed based on an infrared vision system and segmentation algorithms using skin aspect in IR. Images of defects were acquired using a digital monochrome camera with band-pass filters on the near-infrared (NIR). In this case, they do not use visible color and texture, which are necessary in our test. Moreover, they classified the olives individually, and we do it in lots. In [26], the applicability of VIS/NIR spectroscopy was studied as a rapid technique to check SSC (soluble solid content) and texture of olive berries as a quick evaluation of olive fruit ripening index, directly at the mill just before starting the oil extraction process. The results obtained were encouraging, especially regarding the texture estimation. Furthermore, in [27], computer vision was used to analyze the level of impurities in oil samples. They reached good results with success ratios of 87.66% using kernel principal components analysis (KPCA).

To the best knowledge of the authors, there are only a few works that have been used for the automatic selection of olives for oil production and none of them with the goal of differentiating between soil and tree using image analysis as the main technique (thus, they do not consider the inner characteristics of this problem).

## 2. Materials and Methods

The hardware setup, the image processing and the algorithms used for the classification are explained in the following parts.

### 2.1. Experimental Setup

A basic computer vision system was build for the hardware setup. The type of computer was a personal computer (PC) with the MATLAB® acquisition toolbox installed. The vision device was a Logitech QuickCam Sphere AF camera with a resolution of 2 megapixels; it acquires images with 24 bits in depth and 1600 × 1200 pixels in size. The lighting system was a low-angle LED ring with a white diffuser and 16 W of power (INFAIMON®), which provides appropriate measurement conditions.

**Figure 1:**
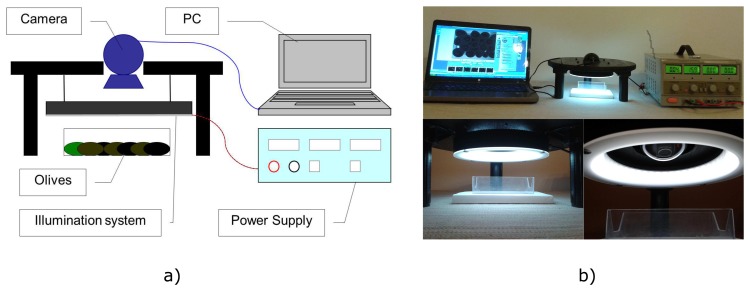
At-line acquisition system setup. (**a**) The setup as a sketch and (**b**) the real setup.

The camera is rigidly attached, as well as the LED ring. A white plastic tray, where the olives are placed before for the acquisition, is placed under the camera on the illumination system. The distance between the camera and the olives can be varied manually. The image processing and the statistical analysis software have been programmed in MATLAB® on Windows SO. Figure 1 shows the prototype used to acquire the images of olives.

The selection and disposition of the illumination system is crucial. We found some problems related to the brightness of the light for the surface of some kinds of fruit. There was too much light, and the intensity of the shine complicated the process to see the defects or wrinkles in the olive skin. The same issue has been reported in other works [22].

**Figure 2:**
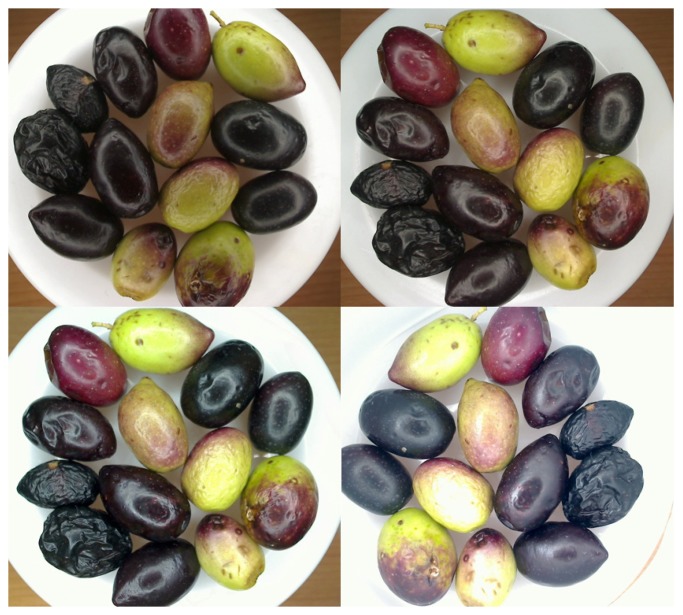
Difference in brightness with different voltages.

We tried to solve the problem in different ways. Firstly, we tested different light sources, two LED rings and a multipoint square light source. We detected that the rings were more suitable for this application. The smaller LED rings 17 cm in diameter were chosen. After this, the light intensity was tested. As a result, the DC power supply was connected to the illumination system in order to change the voltage and the current. That is why the intensity of the light can be modified. Then, the supplied voltage to the illumination system was adjusted in order to avoid the saturation of the pixels over the green olives, because these ones reflect more light than the rest of the olive colors. We supplied a constant voltage of 15.9 V and 0.04 A to the LED ring. This voltage is lower than the nominal voltage of 24 V. Figure 2 shows 4 pictures of the same olives with different light intensities.

**Figure 3:**
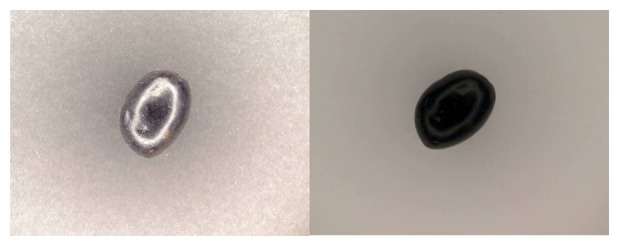
Difference contrasts with different background colors.

Finally, a white background for the plastic tray was selected. Different colors were checked, and a better contrast was obtained using the white color. Figure 3 shows a picture of an olive with two different color backgrounds in white and in black. In these pictures, it is easy to appreciate the differences of contrast and brightness. A white plate was used in other works [23], too.

We also have tested different distances between the light source, the camera and the tray of olives. The adjustment of the position of each element has been necessary in order to see a complete tray of olives and to have the best possible illumination conditions using our computer vision system. Therefore, we have placed the LED ring at a distance of 7 cm from the tray of olives. In addition, the camera was attached to the support with a distance of 9 cm from the olive tray. The plastic tray that contains the olives during the image acquisition is 6.5 cm in width and 10.5 cm long.

### 2.2. Olive Fruit Samples

The experiments were carried out with samples of olives harvested during January and February in 2014, in the region of Priego de Cordoba, Spain. A sample set with 176 olive batches among different varieties (Picudo, Picual and Hojiblanco) was recollected by hand and randomly. Of these, 77 samples were picked from the soil, and 99 samples were picked from the tree. For the objective classification of this work, we have taken into account olives with mid- and advanced maturity indexes because normally, early olives (green ones) do not fall at these stages; therefore, the two classes are not available.

Olive images were obtained under static conditions the same day of the harvest. Each batch contains between 15 to 25 olives depending on the size of olives, and the size of the olive is directly linked to the variety and the agronomic conditions of the tree.

### 2.3. Image Analysis

Different authors have worked with images where the olives have been segmented to find some defects on the fruit surface [28]. We propose the use of entire images for our classification aim instead of segmenting each olive fruit. This proposal is interesting, because undesirable components in the images could provide extra information about the class. Nevertheless, this methodology is not able to identify the olive pieces belonging to each class in the same batch with the objective of classifying each olive fruit individually. However, this ability could be less efficient in terms of time consumption at the industrial scale.

**Figure 4:**
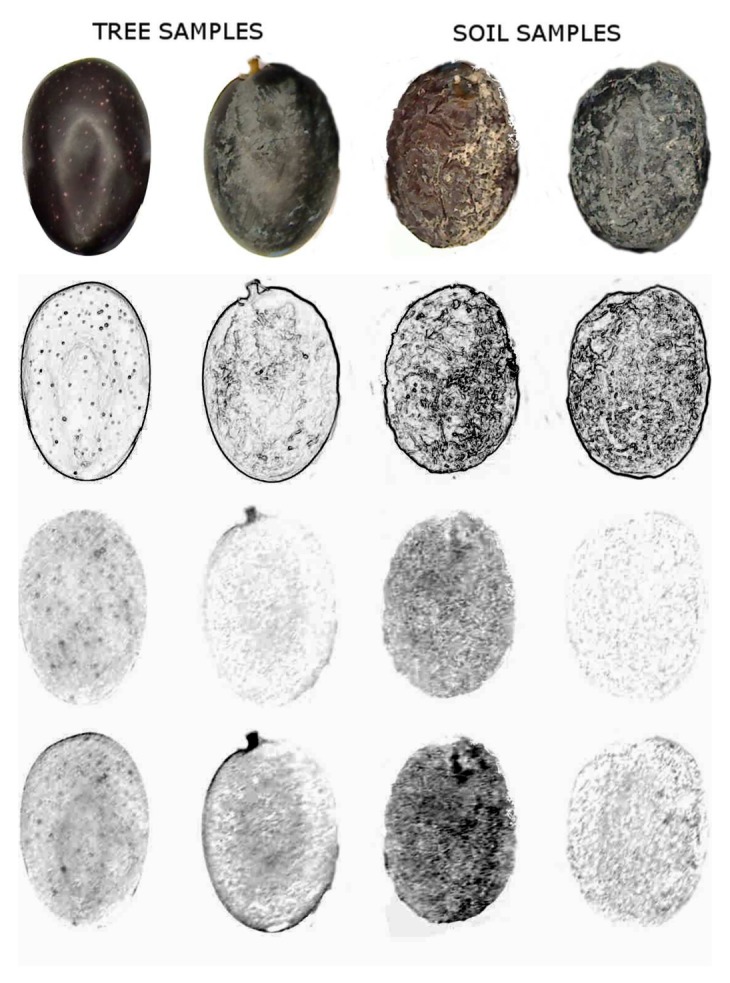
Olives coming from different locations and different image analyses. The first row shows the original image of the samples (RGB); the second row presents the result after applying the textural filter (I), and the third and fourth rows show the same samples where a brownish color has been detected (*C_rg_* and *C_rb_*, respectively).

Two image processing methods were applied: gradient image and differences in the RGB channels [29]. The first one was focused on the detection of sudden changes in the intensity profile of images. This fact is related to detecting olives with wrinkled skin. We can see the results of applying this filter in the second row, as shown in Figure 4, where abrupt changes in the olive skin have been contrasted. The darker they are, the more wrinkled they are. By employing this filter, the differences between tree and soil samples are more obvious.

The gradient image was obtained by applying the textural filter according to Equation (1).

(1) $$\nabla I\left(x,y\right)=G\left(x,y\right)=\sqrt{G_{x}^{2}+G_{y}^{2}}$$

where *I*(*x*, *y*) is the original gray-scale image of each sample and *G*(*x*, *y*) is its gradient image. This gradient image was obtained by using the Roberts operator [30] and by convolving the *I*(*x*, *y*) image with two convolution masks, which are shown in Equations (2) and (3).

(2) $$G_{x}=I\left(x,y\right)*\left[\begin{array}{cc} 1 & 0\\ 0 & -1 \end{array}\right]$$

(3) $$G_{y}=I\left(x,y\right)*\left[\begin{array}{cc} 0 & 1\\ -1 & 0 \end{array}\right]$$

The gradient will be zero in areas with constant intensity levels, and it will be proportional to the intensity level variations in the other ones. In this case, the image processing toolbox of MATLAB® [31,32] was employed.

On the other hand, the brownish color appears in the RGB images when the red channel intensity levels are greater than the green and blue ones. Therefore, the presence of brownish tones in the images was identified with the difference between red and green channels, including the difference between red and blue channels. Thus, two new images *C_rg_*(*x*, *y*) and *C_rb_*(*x*, *y*) are obtained (Equations (4) and (5)).

(4) $$C_{rg}\left(x,y\right)=RGB_{R}\left(x,y\right)-RGB_{G}\left(x,y\right)$$

(5) $$C_{rb}\left(x,y\right)=RGB_{R}\left(x,y\right)-RGB_{B}\left(x,y\right)$$

where RGB is the original image.

The third and fourth rows in Figure 4 show an example of the image analysis previously mentioned for four olives. In this case, dark tones indicate brownish colors, and the differences between soil and tress classes can be observed.

### 2.4. Feature Vector

All samples were translated into feature vectors with the goal to be mathematically usable in the next steps. Before using the classification algorithms, the gray-level histogram of each image (*I*, *C_rg_* and *C_rb_*) was obtained. In order to build the feature vector of each sample, the histograms were concatenated to obtain a vector of 768 components (256 components per image). Each component was centered to have mean of zero and scaled to have a standard deviation of unity.

There are essentially two approaches for feature selection: filter-based feature selection and wrapper-based feature selection. Their differences can be seen in [33]. Stated briefly, the filters evaluate feature subsets by their information content, and the wrappers set the objective function as a pattern classifier. A filter algorithm has been selected due to its fast execution and generality for future samples. The one-way analysis of variance (ANOVA) is like a filter algorithm, where the distance between classes was used to evaluate the difference of 768 components for each olive class. For each component, if the *F*-value is greater than 2, the inter-class distance is the double of the intra-class distance, and we can consider the component as a discriminant feature. Moreover, if the *p*-value is greater than 0.05, the *F*-value is statistically significant by agreement. The optimal components were selected to discriminate among these classes.

Finally, principal component analysis (PCA) [34] was carried out on the resultant histograms to investigate possible clustering of samples on the basis of sample origin. PCA is a linear and unsupervised method helpful for reducing the dimensionality of numerical datasets in a multivariate problem. This method can extract useful information from the data and identify the relation among different classes. The statistic toolbox of MATLAB® [35] was used for the above tasks, and the algorithm to implement PCA was eigenvalue decomposition.

### 2.5. Classification Algorithms

Linear and nonlinear algorithms were compared for classifying among classes. The linear method was Fisher discriminant analysis (FDA) [36], and the nonlinear one was an artificial neural network (ANN) [37]. The accuracy of each method was proven with cross-validation for fold size of 2, that is 50% of samples for the training phase and the other 50% for the validating phase. The 2-fold CV has been chosen because our objective was to avoid overtraining and to maximize the number of validation samples at the same time. This fact will allow us to generalize the results for future samples. The results can be seen in the Experimental Results and Discussions Section.

#### 2.5.1. Fisher Discriminant Analysis

FDA is a simplified case of LDA [38] in which only two classes appear. Both of them are supervised methods and seek to find a new reduced space that maximizes the separation among classes. For two classes, the solution proposed by Fisher was to maximize a function that represents the difference between the means, normalized by a measure of the within-class variability Thus, the Fisher linear discriminant is defined as the linear function (Equation (6)), which maximizes the objective function presented in the Equation (7).

(6) $$y=w^{T}x$$

(7) $$J\left(w\right)=\frac{\left|{\tilde{\mu }}_{1}-{\tilde{\mu }}_{2}\right|}{{{\tilde{s}}_{1}}^{2}+{{\tilde{s}}_{2}}^{2}}$$

where *μ̃*_1_ − *μ̃*_2_ is the difference between the different class means and *s̃*_1_^2^ + *s̃*_2_^2^ measures the variability within the two classes after projection; hence, it is called the within-class scatter of the projected samples.

**Figure 5:**
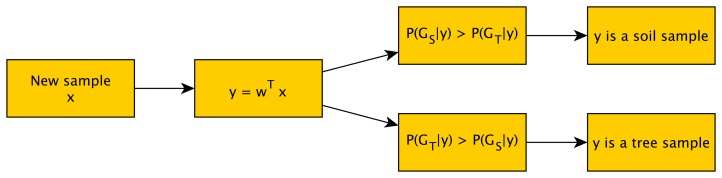
Proposed Fisher discriminant analysis (FDA) classifier diagram.

The next step for this classification algorithm is to fit the multivariate normal density of each group in the new space. Finally, for grading samples, the probability of belonging to each group (GSand GT) is obtained for each sample (Figure 5). The statistic toolbox of MATLAB® was employed, as well.

#### 2.5.2. Artificial Neuronal Network

The selected artificial neural network was multilayer perceptron (MLP) as a nonlinear classification method.

**Figure 6:**
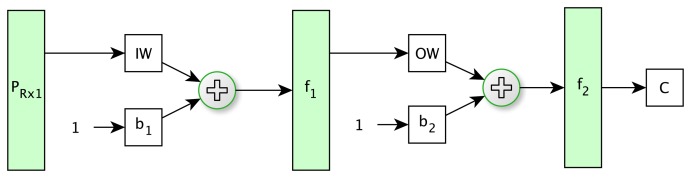
Neuronal network diagram where *P* is the input vector; *IW* is the input weights; *OW* is the output weights; *b*_1_ and *b*_2_ are the bias; *f*_1_ and *f*_2_ are the sigmoid functions; and *C* is the classification result.

Only one hidden layer was considered, since this configuration is capable of learning any pattern and less prone to getting caught in local minima in those networks with a higher number of hidden layers [39]. The number of input nodes was the same as vector components selected in the previous step. Furthermore, the neural network was tested with different numbers of neurons in the hidden layer. The classification results were similar; therefore, one neuron was established. Finally, the output layer was configured with one neuron, because it is sufficient to classify between soil and tree classes. Figure 6 shows the net topology that has been employed.

The ANN was trained with the backpropagation algorithm named scaled conjugate gradient [40], because it has been proven to be efficient in pattern recognition problems [41]. In contrast, the activation function for the hidden layer was sigmoidal in order to learn nonlinear relationships between input and output vectors. Additionally, the activation function of the output layers was sigmoidal to constrain the network output. In this case, the Neural Network toolbox of MATLAB® was used [42].

## 3. Experimental Results and Discussions

**Figure 7:**
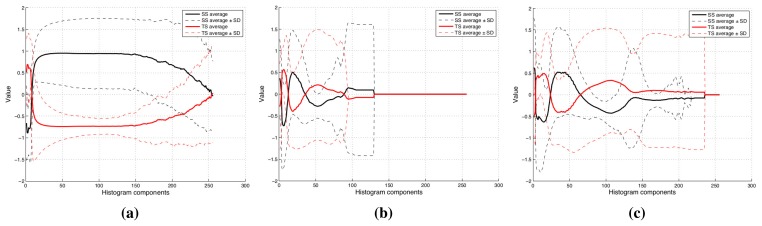
This figure shows the normalized image histograms after applying the textural filter (**a**), channel R minus channel G operation (**b**) and channel R minus channel B operation (**c**).

The histograms of the processed images for the whole dataset can be seen in Figure 7a–c. The average values for each component and their standard deviation appear in this figures. The difference between two olive classes is more evident in the textural image histograms (Figure 7a) than in the other ones. This fact proves that the wrinkling of the olive surface is an important factor to arrange classes. However, in the other histograms (Figure 7b,c), several components appear that could be interesting in order to build the feature vector.

**Figure 8:**
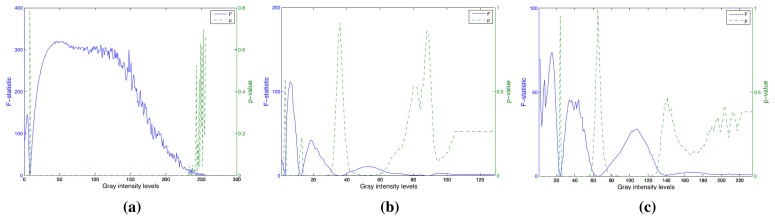
This figure shows the statistical *F*-values and *p*-values between the aforementioned two classes of olives for the histograms in Figure 7.

The variance analysis ANOVA was carried out in order to search the histogram components and more discriminants between classes. The results of this test for each component are shown in the Figure 8a–c. The *F*-value indicates the distance between two olive classes, and the *p*-value is the significant parameter. In these figures, we can see that *F*-values in the texture histogram are in general greater than color histogram ones. As a result, we can confirm the high weight of the textural information in the classification task.

**Figure 9:**
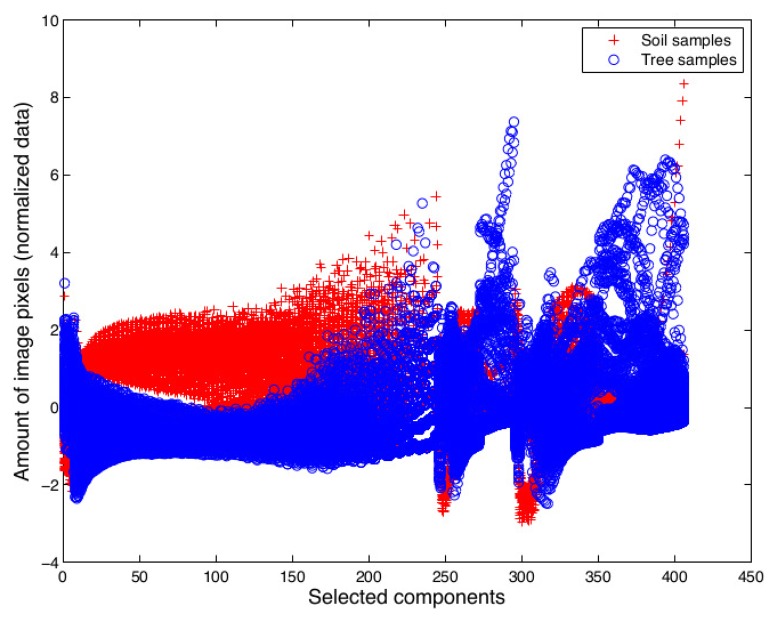
Feature vectors of the whole dataset after filtering by the ANOVA test.

Then, for building the feature vector, the component was excluded if its *p*-value is higher than 0.05 and its *F*-value is lower than two, indicating that the gray intensity level of the histogram has little or no influence on the discrimination of classes. In contrast, if the *p*-value is lower than 0.05 and its *F*-value is higher than two, the histogram component is included in the feature vector. Finally, the number of 768 components was reduced to 405 significant components. The feature vector for the whole dataset is shown in Figure 9.

**Figure 10:**
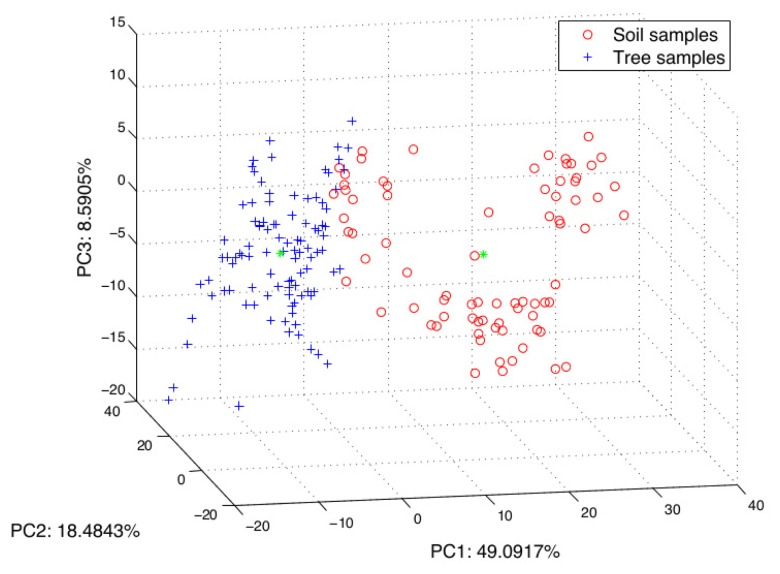
All of the dataset is projected in the new 3D space, which is created by three principal components, PC1, PC2 and PC3.

The next step was to eliminate co-linearity and to reduce the amount of components in the feature vector. The clustering among classes is evidenced from the results obtained by PCA applied to the matrix of the entire set of scaled histogram data. Figure 10 shows the samples projected in the three firsts components. In this case, the explained variance was about 75%.

Table 1 shows the results for the two classifiers considered. The second column in the table presents the algorithms followed to process the feature vectors of each sample, where NORM means normalization; ANOVA is the feature selection algorithm, and PCA is the feature extraction algorithm. These algorithms have been explained in the Materials and Methods Section. The third column shows the image information used to assemble the feature vector, where TEXT is textural information, RG is red and green channel and RB is red and blue channel. Finally, it shows the classification results for a number of PCA components.

The dataset was randomly divided in two groups with the same size for training and validating purposes. The correct classification percentage was the average of two iterations according to 2-fold cross validation. For each iteration, this percentage was obtained from dividing the number of samples classified well in each class (tree and soil) by the amount of samples in the dataset (Equation (8)). Since the dataset was randomly divided into two groups, the aforementioned validation process was repeated three times, and the average value was selected.

(8) $$\text{CCP}=\frac{\text{sum}\left(\text{diag}\left(CM\right)\right)}{TS}\times 100$$

were CCP is the correct classification percentage, CM is the confusion matrix and TSis the total number of samples.

**Figure 11:**
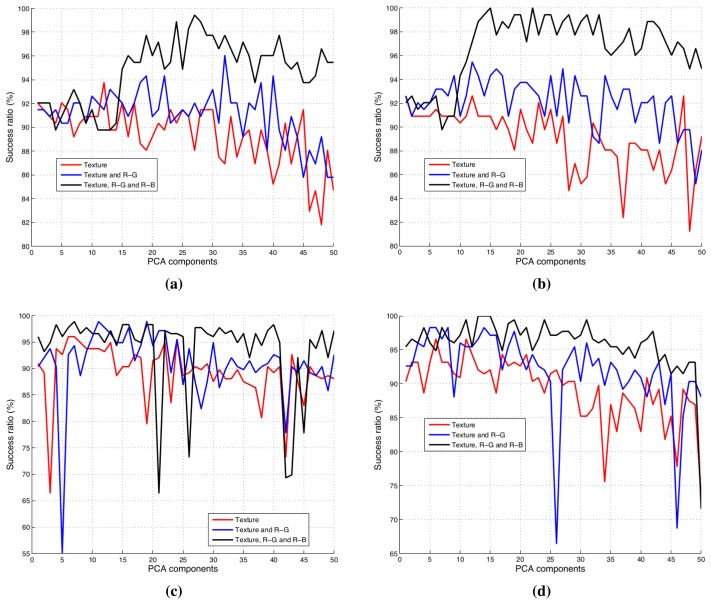
(**a**) Linear classification results without the ANOVA component filter; (**b**) linear classification results with the ANOVA component filter; (**c**) ANN classification results without the ANOVA component filter; (**d**) ANN classification results with the ANOVA component filter.

On the other hand, the classification results obtained with the linear classification algorithm (FDA) denote that it is a good approach to classify olive batches. These results were expected, because the PCA scores with the three principal components are almost separable with a plane (see Figure 11). In this case, the best result was reached with 15 PCA components, the three channels and the feature selection algorithm.

The correct classification result was also 100% with the neuronal network. Nevertheless, ANN obtains this percentage with a lower value of PCA components. This means that the data are linearly separable, but the neural network could be more successful with future samples.

On the other hand, we can see that ANOVA filtering is useful because it reduces the number of PCA components for the same success ratio. In addition to this, this ratio is slightly decremented in both classifiers with only the textural information of the samples that is used. Nonetheless, these results are improved when the color information of the sample images is added. This fact justifies the use of both histograms.

Moreover, the optimal number of PCA components was selected with an iterative process: firstly, the PCA components were ordered by importance (according to the explained variance); for each iteration, a new PCA-component was added, and the sorting accuracy was obtained. Figure 11 shows the results of this iterative process for the nine cases in Table 1. In this figure, we can observe that when the number of component increases, the accuracy of the classifiers decreases because of the overfitting. On the contrary, the performance of the ANN is better than FDA with the first PCA components being employed. Then, we can reduce the number of features without high losses, and the computation time could be improved. This margin could be positive in order to use the classification system in an industrial classification machine of olive batches.

## 4. Conclusions and Future Work

This paper presents the intelligent block of an automatic classifying system of olives based on computer vision before being processed for oil extraction. Different problems have been solved, such as the brightness produced by the olive skin or the hardware setup. This system is able to distinguish between soil and tree olives batches for olive oil production, and it uses information, such as the wrinkles of their skin and their colors. The grading algorithms proposed are linear discriminant analysis and neuronal networks. The goodness of the results (success ratios of 100%) has allowed us to start different tests in a real plant with the building of an automatic sorting machine as a final objective. What is more, this fact has awoken the interest of different manufacturing companies of olive mill machinery.

As future work, it could be interesting to test the same classifier using olives with different ripening indexes and to prove the robustness of the algorithm with batches of previously olives cleaned (which reduces the dirt on the skin of the olives). To improve the classification, a good idea could be taking into consideration other features, such as the difference in olive sizes. Furthermore, the detection of batches of olives with diseases and other problems, such as frozen or mixed olives, could also be an interesting work to be done.

There is another category of olive batches in mills called mixed olives, being a mixture of olives coming from the soil and from the tree in the same lot. It could be very interesting for future work to study the classifier testing lots of mixed olives for different percentages of olives coming from the soil or the tree.

Last, but not least, the classification of the olives individually could be also interesting as future work.

## Figures and Tables

**Table 1:** Classification results. NORM, normalization; TEXT, textural information.

**Classifier**	**Feature Vector Processing**	**Image Information Used**	**Number of PCA Components**	**Correct Classification**
FDA	NORM+ PCA	TEXT	12	93.75%
		TEXT + RG	19	94.32%
		TEXT + RG +RB	27	99.43%
	NORM + ANOVA+ PCA	TEXT	12	92.61%
		TEXT + RG	12	95.45%
		TEXT + RG +RB	15	100%

ANN	NORM+ PCA	TEXT	11	98.86%
		TEXT + RG	6	96.02%
		TEXT + RG +RB	7	98.86%
	NORM + ANOVA+ PCA	TEXT	6	96.60%
		TEXT + RG	5	98.30%
		TEXT + RG +RB	13	100%
